# Deep learning for automated organ activity profiling in sarcoidosis

**DOI:** 10.1093/bib/bbaf631.052

**Published:** 2025-12-12

**Authors:** Sonja Katz, Bowen Fan, Michael Krauthammer, Jakob Nilsson

**Affiliations:** Department of Immunology, University Hospital Zurich, Switzerland; University of Zurich, Switzerland; University of Zurich, Switzerland; Department of Immunology, University Hospital Zurich, Switzerland

## Abstract

**Background:**

Sarcoidosis is a complex, multisystem inflammatory disease with variable organ involvement and course^1^. ^18^F-FDG PET/CT is the gold standard for activity assessment but is costly, scarce, and operator-dependent.

**Methods:**

We present a fully automated deep-learning pipeline for multi-organ PET/CT quantification, applied to 180 patients with 770 longitudinal scans linked to clinical and laboratory data. Based on contrast-enhanced CT, the lungs, myocardium, spleen, and liver are segmented using TotalSegmentator^2^; co-registered PET images provide organ-level SUV_max_ values and metabolic activity scores.

**Results:**

Pipeline-derived metrics showed strong agreement with clinician assessments of cardiac involvement (for activity, r = 0.93; for SUV_max,_ r = 0.95, both p < 0.001). Organ-resolved analyses revealed significant correlations between PET metrics and inflammatory biomarkers, particularly in lung involvement (e.g., serum TNF-α, r = 0.39, FDR < 0.05). Using repeated measurements of disease activity and biomarker concentrations, longitudinal modeling captured flare–remission dynamics. Biomarker trajectories paralleled PET activity, supporting their potential as non-invasive surrogate markers when PET/CT is unavailable; in practice, routine lab tests between imaging visits could flag incoming flares and guide early treatment adjustments.

**Conclusion:**

This automated, organ-resolved PET/CT pipeline, paired with routine labs, enables objective, scalable sarcoidosis profiling beyond tertiary clinics, facilitating early and precise treatment response monitoring and prognostication.

**References:**

1. Grunewald J., Grutters J.C., Arkema E.V., Saketkoo L.A., Moller D.R., Müller-Quernheim J. ‘Sarcoidosis.’ Nature Reviews Disease Primers 2019;5(1):45.

2. Wasserthal J., Breit H.C., Meyer M.T., Pradella M., Hinck D., et al. ‘TotalSegmentator: robust segmentation of 104 anatomic structures in CT images.’ Radiology: Artificial Intelligence 2023;5(5):e230024.

